# Evaluation of hepatoprotective activity of *Cleome viscosa* Linn. extract

**DOI:** 10.4103/0253-7613.48892

**Published:** 2009-02

**Authors:** Nishant Kumar Gupta, Vinod Kumar Dixit

**Affiliations:** Department of Pharmaceutical Sciences, Dr. H. S. Gour Vishwavidyalaya, Sagar, MP, India

**Keywords:** Carbon tetrachloride, *Cleome viscosa* Linn, hepatoprotective

## Abstract

**Objectives:**

To evaluate the hepatoprotective activity of ethanolic extract of *Cleome viscosa* Linn. (Capparidaceae) against carbon tetrachloride (CCI_4_) induced hepatotoxicity in experimental animal models.

**Materials and Methods:**

Leaf powder of *Cleome viscosa* was extracted with ethanol. The hepatoprotective activity of the extract was assessed in CCI_4_ induced hepatotoxicity in rats. Various biochemical parameters were estimated and histopathological studies were also performed on rat liver. The hepatoprotective activity was also supported by determining a functional parameter, i.e. thiopental-induced sleep of mice poisoned with CCl_4_.

**Results:**

The test material was found effective as hepatoprotective, through *in vivo* and histopathological studies. The extract was found to be effective in shortening the thiopental induced sleep in mice poisoned with CCl_4_. The hepatoprotective effect of ethanolic extract was comparable to that of silymarin, a standard hepatoprotective agent.

**Conclusion:**

The results of the present study show that ethanolic extract of *Cleome viscosa* has significant hepatoprotective activity.

## Introduction

*Cleome viscosa* Linn. (Capparidaceae) is a widely distributed herb with yellow flowers and long slender pods containing seeds. The whole plant is sticky in nature and has a strong odour resembling asafoetida. It is found throughout the greater part of India, often in waste places and is known as Hurhur (Hindi) in Indian traditional medicine. Traditionally, this plant is used in various disorders such as diarrhoea, fever, inflammation, liver diseases, bronchitis, skin diseases, and malarial fever. The juice is useful in piles, lumbago and earache.[1] The analgesic, antipyretic and anti-diarrhoeal activities of the extract have been reported by researchers.[2–4] In our field studies of ethno-medicinal plants of Bundelkhand, it was noted that the fresh leaves of *Cleome viscosa* are widely used as medicine for jaundice. A systematic study to assess the action of the plant for liver diseases has not been reported and hence the present investigations were undertaken.

## Materials and Methods

### Plant material

The plant *Cleome viscosa* Linn. was collected from the forest, in the month of September, 2005 and identified at the Department of Pharmaceutical Sciences, Dr. H. S. Gour Vishwavidyalaya, Sagar. The voucher specimen was deposited in the institute's herbarium (CV 04101). Leaves of the plant were dried in the shade and powdered.

### Chemicals

Carbon tetrachloride (CCl_4_) was purchased from Qualigens Fine Chemicals, Mumbai. Thiopental sodium was purchased from Sigma chemical company. The following were purchased from Bayer Diagnostics India Limited: SGOT, SGPT, Alkaline phosphatase (SALP) and bilirubin (SBLN) kits. Silymarin was a gift from Micro Labs Limited, Goa, India.

### Animals

Male albino rats (Druckrey strain) of 100-150 g and Swiss mice (20-25 g) were obtained from the Central Drug Research Institute, Lucknow, India. The animals were acclimatized to in-house conditions and were fed a commercial pellet diet (Hindustan Lever Limited, Bangalore, India) and water *ad libitum*. Experimental protocol was undertaken in accordance with the ethical guidelines, and the permission of the institutional animal ethics committee was obtained.

### Preparation of extract

500 grams of powdered leaves of the plant were defatted with petroleum ether (60-80°C) and then extracted with a sufficient volume of ethanol for 48 h in a soxhlet apparatus. The extract was filtered and concentrated under reduced pressure and low temperature (40-50°C) on a rotary evaporator (Yield 7.32 % w/w). This part of the test sample was named ethanolic extract.

10 g ethanolic extract was dispersed in 200 ml of distilled water and extracted with ethyl acetate (200 ml) in a separating funnel. Ethyl acetate fraction was separated and concentrated under reduced pressure and low temperature (40-50°C) in a rotary evaporator (Yield 0.732 % w/w). This part of the test sample was named fraction A.

### Carbon tetrachloride induced hepatotoxicity model

Hepatoprotective activity was evaluated by the method described by Saraf and Dixit.[5] The rats were divided into six groups of six rats each. Group A served as normal and were given only vehicle (distilled water, 1ml/kg b.w.) for four days. Group B served as toxin control and was administered with vehicle on the first and fourth day and with vehicle + CCl_4_ (50% solution of CCl_4_ in olive oil, 2 ml/kg b.w.) on the second and third day. Groups C and D received ethanolic extract 100 mg/kg b.w. and 200 mg/kg b.w. on the first and fourth day and ethanolic extract (100 and 200 mg/kg b.w.) + CCl_4_ on the second and third day, respectively. Group E received fraction A (10 mg/kg b.w.) on the first and fourth day and fraction A (10 mg/kg b.w.) + CCl_4_ on the second and third day. Group F received silymarin (50 mg/kg b.w.) on the first and fourth day and silymarin (50 mg/kg b.w.) + CCl_4_ on the second and third day. Administration of CCl_4_ was intra-peritoneal, whereas the extracts, fraction and silymarin were given orally.

### Assessment of hepatoprotective activity

On the fifth day, the animals were anaesthetized and blood was collected from the retro-orbital plexus. Serum was separated after coagulating at 37°C for 30 min and centrifuging at 2000 rpm for 15 min, and estimated for serum glutamate oxaloacetate transaminase (SGOT), serum glutamate pyruvate transaminase (SGPT),[6] alkaline phosphatase (ALKP)[7] and serum bilirubin (SBLN)[8] using kits supplied by Bayer Diagnostics India Limited.

The hepatoprotective activity was confirmed through histopathological studies on liver of rats. After collection of blood for biochemical estimation, the rats were sacrificed and the liver was carefully dissected, cleaned of extraneous tissue, and fixed in 10% formalin for at least 24 h. Then the paraffin sections were prepared (automatic tissue processor, Autotechnique) and cut into 5 *μ*m thick sections, using a rotary microtome. The sections were stained with Haematoxylin-Eosin dye and studied for histopathological changes.[9]

### Barbiturate sleeping time

The hepatoprotective activity was also supported by determining a functional parameter, i.e. thiopental-induced sleep of mice poisoned with CCl_4_.

The time of lost reflex induced by short acting barbiturate (thiopental sodium) is significantly prolonged in the event of hepatic damage and can be used as a measure of the functional status of the drug metabolizing enzymes in the liver.[10–12] Thirty-six Swiss mice were selected and randomly divided into six groups of six each. Group A was kept as normal and normal sleeping time was determined after injecting the thiopental sodium (25 mg/kg b.w.). Group B was kept as control and administered CCl_4_ (1 ml/kg b.w.). Group C and D were treated with ethanolic extract 100 mg/kg b.w. and 200 mg/kg b.w. respectively. Group E and F were given a dose of fraction A (10 mg/kg b.w.) and silymarin (50 mg/kg b.w.) respectively. After 24 h, a dose of CCl_4_ (1 ml/kg b.w.) was given to B, C, D, E and F groups. Sleeping time was determined against thiopental sodium injection (25 mg/kg b.w.) after two hours of CCl_4_ injection. CCl_4_ was administered intra-peritoneally, whereas the extracts, fraction and silymarin were given orally.

### Statistical analysis

Results are presented as mean ± standard error (S.E.) of mean and percentage degree of reversal against hepatotoxin. Percentage was calculated by considering enzyme level difference between CCl_4_ and normal rats as 100% degree of reversal. The data were analyzed for significance, using the unpaired two-tailed Student's *t*-test.[13]

## Results

### In vivo hepatoprotective activity

Results presented in Table 1 indicate that the elevated levels of SGOT, SGPT, ALP and BLN (total and direct) due to CCl_4_ intoxication were reduced significantly (*P*<0.001) in rats, after treatment with ethanolic extract. Treatment with ethanolic extract at a dose of 100 mg/kg b.w. decreased the SGOT, SGPT, ALP, BLN (direct and total) levels by 55.55, 54.08, 44.08, 86.55 and 83.81%, respectively, while a higher dose of 200 mg/kg b.w. was more effective, causing a reduction of 84.20, 78.04, 79.99, 96.64 and 99.78%. Silymarin used as positive control showed a reduction of 89.94, 87.45, 81.36, 89.92 and 84.04% respectively.

**Table 1:** Effect of *C. viscosa* on serum parameters of CCl_4_-treated rats

*Group*	*SGOT (U/l)*	*SGPT (U/l)*	*SALP (U/l)*	*Direct bilirubin (mg/dl)*	*Total bilirubin (mg/dl)*
A. Normal	97.16 ± 2.51	40.33 ± 2.51	66.83 ± 2.65	0.18 ± 0.03	1.03 ± 0.04
B. Toxin control (CCl_4_)	239.67 ± 5.16	173.17 ± 3.42	152.67 ± 3.20	1.37 ± 0.09	5.54 ± 0.16
C. Ethanolic extract (100 mg/kg b.w.) + CCl_4_	160.50 ± 3.09 (55.55)	101.33 ± 2.39 (54.08)	114.83 ± 2.13 (44.08)	0.34 ± 0.02 (86.55)	1.76 ± 0.25 (83.81)
D. Ethanolic extract (200 mg/kg b.w.) + CCl_4_	119.67 ± 2.51 (84.20)	69.50 ± 2.62 (78.04)	84.00 ± 1.95 (79.99)	0.22 ± 0.02 (96.64)	1.04 ± 0.11 (99.78)
E. Fraction A (10 mg/kg b.w.) + CCl_4_	242.67 ± 3.71^a^ (- 2.11)	171.17 ± 3.77^a^ (1.51)	139.67 ± 3.42^a^ (15.14)	1.02 ± 0.06^a^ (29.41)	4.90 ± 0.11a (14.19)
F. Standard (Silymarin, 50 mg/kg b.w.) + CCl_4_	111.50 ± 3.21 (89.94)	57.00 ± 2.03 (87.45)	82.83 ± 2.21 (81.36)	0.30 ± 0.02 (89.92)	1.75 ± 0.09 (84.04)

Values are means ± S.E.M, *n* = 6. Values in Parenthesis indicate percentage recovery.

Values are statistically significant at *P*<0.001 compared to group B.

a *P*<0.001 compared to group A.

### Barbiturate sleeping time

Results presented in [Table 2] indicate that both ethanolic extract groups (100 and 200 mg/kg b.w.) have been able to significantly (*P* < 0.001) reduce thiopental “sleeping-time” in mice (50.66 and 83.55%, respectively), as compared to animals receiving CCl_4_ alone. The fraction A was not able to reduce the sleeping-time, as compared to the toxin control (CCl_4_) group.

**Figure 3c:**
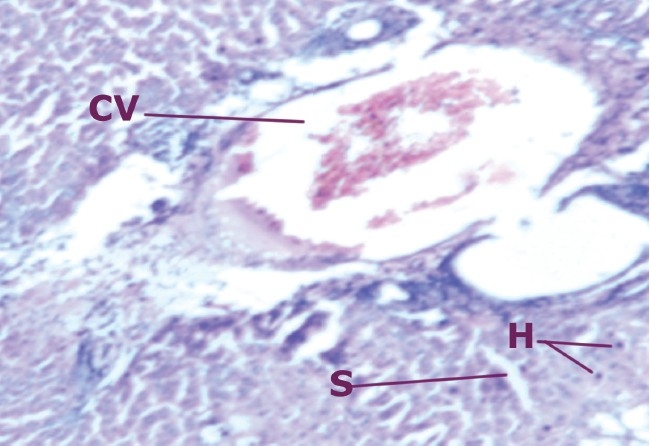
Liver cells of rats treated with fraction A and intoxicated with CCl_4_

**Figure 4:**
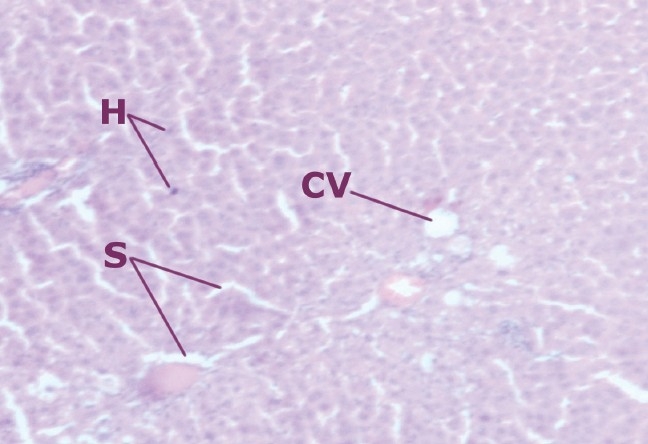
Liver cells of rats treated with silymarin and intoxicated with CCl_4_

### Histopathological studies

**Figure 1:**
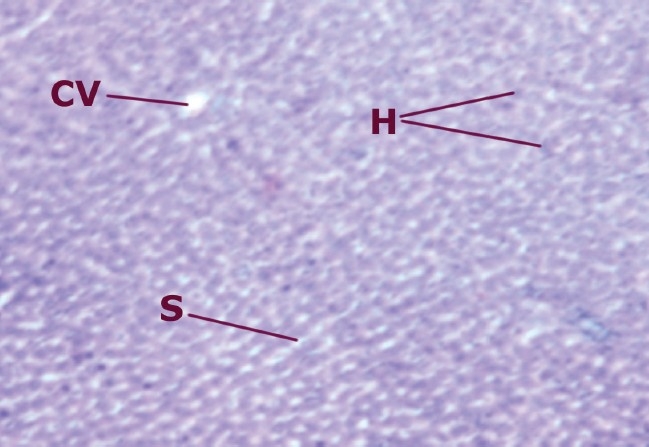
Liver cells of normal rats

**Figure 2:**
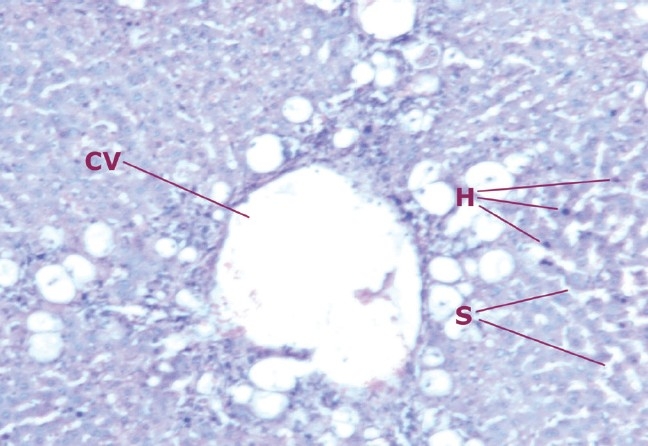
Liver cells of rats intoxicated with CCl_4_

A comparison of the liver section of animals treated with CCl_4_ showed a high degree of damage characterized by cell vacuolation, pyknotic and degenerated nuclei and wall of bile capillaries. The normal architecture of the liver was lost. The intralobular vein was badly damaged with wide spaces at some sinusoids [Figure 1–2].

In the liver section of the animals treated with ethanolic extract (100 mg/kg b.w.) + CCl_4_, the nucleii are not very clear as in normal hepatocytes; however, when compared to the CCl_4_ damaged group, the number of hepatocytes with normal nucleus was much more. The endothelium is disrupted in places. Pyknotic nucleus and vacuolation in cytoplasm are observed to be low, as compared to the CCl_4_ group [Figure 3a].

**Figure 3a:**
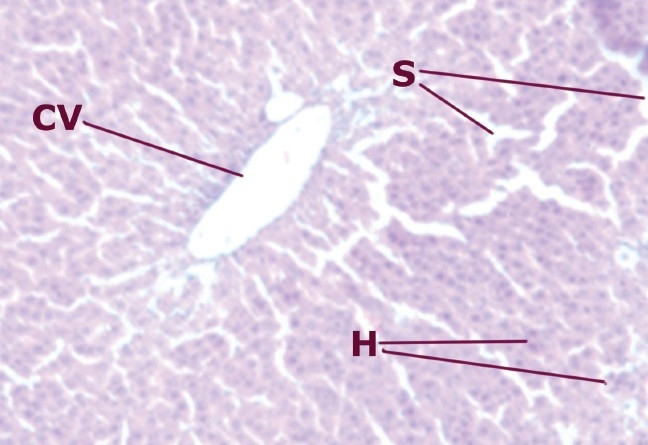
Liver cells of rats treated with ethanolic extract (100 mg/kg b.w.) and intoxicated with CCl_4_

The hepatic cells of rats treated with ethanolic extract (200 mg/kg b.w.) and intoxicated with CCl_4_ were radially arranged. The vacuolation was present, similar to that of normal. The recovery was comparable to that with silymarin, a standard hepatoprotective agent. The intralobular vein was normal in structure, but the wall was damaged, as shown in Figure 3b. No pyknosis in the nucleus was seen.

**Figure 3b:**
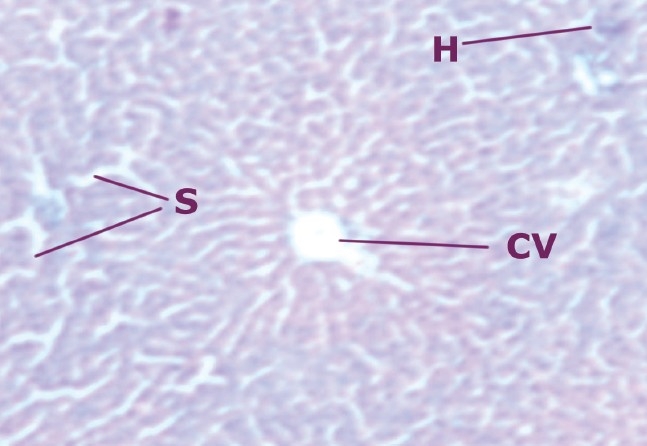
Liver cells of rats treated with ethanolic extract (200 mg/kg b.w.) and intoxicated with CCl_4_

Liver sections of rats that were treated with fraction A along with CCl_4_ exhibited a high degree of damage suggesting negligible protection to the liver by fraction A.

## Discussion

The presence of jaundice is a cardinal feature of liver disease and its presence usually shows disturbance involving the hepatobiliary system.[14] Carbon tetrachloride is a potent hepatotoxin, and a single exposure to it can rapidly lead to an increase in the level of several enzymes, severe centrizonal necrosis and steatosis.[15] The hepatotoxicity induced by CCl_4_ is due to its metabolite CCl_3_•, a free radical which binds to lipoprotein and leads to peroxidation of lipids of the endoplasmic reticulum.[16] The ability of a hepatoprotective drug to reduce the injurious effects or to preserve the normal hepatic physiological mechanisms, which have been disturbed by a hepatotoxin, is the index of its protective effects. The lowering of enzyme level is a definite indication of the hepatoprotective action of the drug. Protection of hepatic damage caused by carbon tetrachloride administration was observed by recording SGOT, SGPT, SALP and SBLN levels in different groups.[17] The transport function of the hepatocytes is disturbed in hepatic injury, causing the leakage of enzymes due to altered membrane permeability.[18]

Although both the doses of ethanolic extract offer hepatoprotection, the higher dose (200 mg/kg b.w.) is more effective [Tables 1 and 2]. Depending on the type of cell and the membrane involved, lipoperoxidation due to CCl_4_ results in hemolysis, which increases the serum bilirubin level.[16] The 200 mg/kg dose of extract showed an effective derange in the elevated serum bilirubin level, as compared to the low dose. The extract thus has a potential application in the acute condition of jaundice. A possible mechanism of the extract on bilirubin levels may be interference with cytochrome P_450_, resulting in the hindrance of the formation of hepatotoxic free radicals, thereby protecting the integrity of the membrane.

The ability of the ethanolic extract of *Cleome viscosa* Linn. to reduce the prolongation of thiopental-induced sleep in CCl_4_ poisoned rats is further indicative of the hepatoprotective potential of the test substance. It has been established that since the barbiturates are metabolized almost exclusively in the liver, the sleeping time after a given dose is a measure of hepatic metabolism.[12] If there is any liver damage, as in this case by CCl_4_ intoxication, the sleeping time after a given dose of the barbiturate will be prolonged, because the amount of the barbiturate metabolized per unit time will be less.

The histopathological studies also exhibit the efficacy of drug as a hepatoprotective. Simultaneous treatment of ethanolic extract with CCl_4_ produces lesser degree of damage to the liver cells as compared to the animals treated with CCl_4_ alone. The sections of the liver treated with extract (200 mg/kg b.w.) and CCl_4_ reveal better hepatoprotective activity almost similar to the standard (silymarin) group. Negligible damage to a few hepatocytes present in the close vicinity of the intralobular vein is observed. Hepatocytes show normal appearance with the presence of vacuoles in the cytoplasm.

These data, along with the histopathological studies, clearly show the hepatoprotective activity of *Cleome viscosa* Linn. and justify the use of this plant for jaundice.
